# Real-world data of opicapone in patients with Parkinson’s disease experiencing motor fluctuations: the OPTIMO study

**DOI:** 10.3389/fneur.2026.1738500

**Published:** 2026-02-19

**Authors:** María‑Rosario Luquin, Nuria Lopez-Ariztegui, Juan Carlos Martínez Castrillo, Lydia López Manzanares, Isabel Sastre Bataller, Antonio Koukoulis Fernández, Bárbara Vives Pastor, Berta Solano Vila, María Álvarez Sauco, Javier Pagonabarraga Mora, José Matías Arbelo González, Pedro José García Ruiz-Espiga, Oriol de Fàbregues, Javier López del Val, Víctor Campos Arillo, Clara Moreno, José Blanco Ameijeiras, Isabel Pijuan Jiménez, Iciar Tegel Ayuela

**Affiliations:** 1 Clínica Universidad de Navarra, Pamplona, Spain; 2 Hospital Universitario de Toledo, Toledo, Spain; 3 Servicio de Neurología, IRYCIS, Hospital Universitario Ramón y Cajal, Madrid, Spain; 4 Hospital Universitario La Princesa, Madrid, Spain; 5 Hospital Universitario y Politécnico La Fe, Valencia, Spain; 6 Hospital Álvaro Cunqueiro, Vigo, Spain; 7 Hospital Universitario Son Espases, Palma de Mallorca, Spain; 8 Hospital Josep Trueta, Girona y Hospital Santa Caterina Salt, Girona, Spain; 9 Hospital General Universitario de Elche, Elche, Spain; 10 Hospital de la Santa Creu i Sant Pau, Institut de Recerca, Barcelona, Spain; 11 Hospital Universitario San Roque, Las Palmas de Gran Canaria, Spain; 12 Hospital Universitario Fundación Jiménez Díaz, Madrid, Spain; 13 Hospital Universitario Vall d’Hebron, Barcelona, Spain; 14 Clínica HLA Montpellier, Zaragoza, Spain; 15 Hospital Vithas-Xanit, Benalmádena, Málaga, Spain; 16 Medical Department, Laboratorios Bial S.A, Madrid, Spain

**Keywords:** catechol-o-methyltransferase inhibitors, motor fluctuations, opicapone, Parkinson’s disease, real-world data, wearing-off

## Abstract

**Purpose:**

Evaluate the outcomes of opicapone as an add-on treatment to levodopa/DDCI in patients with Parkinson’s disease (PD) and motor fluctuations (MF) in a real-world setting.

**Methods:**

Observational, retrospective, and post-authorization study in patients with PD and MF treated with opicapone at 16 Spanish Movement Disorders centers.

**Results:**

Of 245 patients included (55.9% men; mean [standard deviation] age: 67.7 [10.4] years), 41.9 and 33.6% presented rigid-akinetic and tremor-dominant phenotypes, respectively; 43.8% had a history of dyskinesias. Patients started treatment with 50 mg/day opicapone 8.3 (5.3) years after diagnosis. At initiation, the mean levodopa dose was 620.7 (313.7) mg/day. According to the PGI-C (available in 178 patients), 74.2% of patients reported clinical improvement in MF, without worsening of dykinesias in 64.6%. Clinical improvement of MF with stable/improved dyskinesias was similar between PD phenotypes (*p* = 0.327). Opicapone reduced the percentage of patients experiencing wearing-off phenomena (98.0% vs. 61.6%), delayed-on (10.2% vs. 5.3%; *p* = 0.010), no-on (6.5% vs. 2.9%; *p* = 0.027), and non-motor fluctuations (21.6% vs. 15.1%; *p* = 0.010). Furthermore, the off-time decreased (143.3 vs. 67.9 min/day; *p* < 0.001). After 4.8 (3.6) months of treatment, scores in UPDRS Parts II-IV significantly decreased, suggesting additional improvements in daily activities and motor function. The mean daily time with dyskinesias did not increase after initiating opicapone. Mild adverse events were observed in 21 (8.3%) patients.

**Conclusion:**

This study demonstrates that opicapone added to levodopa improves motor function and reduces MF without significantly enhancing dyskinesia intensity, along with a tolerable profile. Moreover, there were no differences regarding clinical improvement among PD phenotypes.

## 1 Introduction

Since the introduction of levodopa for the treatment of Parkinson’s disease (PD), patients’ quality of life has dramatically improved, and nowadays, levodopa associated with a decarboxylase inhibitor (levodopa/DDCI) remains the gold standard treatment for PD (1, 2). However, long-term levodopa therapy along with disease progression induces the appearance of motor and non-motor complications, including on/off motor fluctuations and dyskinesias, which negatively impact patients’ quality of life (3–5). The most common motor complication is the “wearing-off” phenomenon, where PD symptoms reappear just before the next levodopa dose (6). Managing these fluctuations involves adjusting levodopa doses, using different levodopa formulations, or adding drugs that enhance the striatal dopamine tone, like dopamine agonists, monoamine oxidase B (MAO-B) inhibitors, and catechol-O-methyl transferase inhibitors (COMTi) (7, 8).

Opicapone, a third-generation COMTi, was approved in 2016 by European regulatory agencies as an adjunctive therapy to levodopa/DDCI (9). Due to its high binding affinity and slow dissociation rate, opicapone provides long-lasting COMT inhibition with convenient once-daily administration (10, 11). Pharmacokinetic data showed that opicapone add-on levodopa provides higher levodopa bioavailability with avoidance of trough levels, resulting in reduced pulsatile dopaminergic stimulation produced by individual and repeated levodopa doses, thus allowing the use of lower doses of levodopa (12).

The efficacy and safety of opicapone have been consistently demonstrated in two phase III, randomized, placebo-controlled trials, BIPARK I and BIPARK II. These studies showed that opicapone significantly prolonged the clinical effects of levodopa, thereby improving motor function and reducing the daily “off” time while being generally well-tolerated (13, 14). These observations were later supported by some real-world data (RWD) studies (15, 16), such as the OPTIPARK trial that demonstrated the effectiveness of opicapone and improved quality of life in patients with PD after 3 months of routine treatment in clinical practice in Germany and the UK (15). More recently, new data have been published on the efficacy and safety of opicapone when administered with daily doses of levodopa of ≈400 mg in patients who suffered early motor fluctuations (<2 years of motor fluctuations). This randomized, open-label, phase IV clinical study proved that opicapone is more effective in reducing the daily off time than increasing 100 mg of levodopa (16).

Despite the robust clinical evidence supporting the efficacy and safety of opicapone (13, 14), and the availability of RWD on its use (15, 17–19), the present study provides a unique approach as the first comprehensive real-world evidence analysis in Spain, which aims to identify predictive factors for clinical response to opicapone in PD. The OPTIMO study evaluates the effectiveness of opicapone in reducing motor fluctuations in real-life patients with PD, as well as its tolerability and clinical management.

## 2 Methods

### 2.1 Study design and patients

OPTIMO was a multicenter, observational, retrospective, and post-authorization study designed to describe the clinical change derived from opicapone as an add-on treatment to levodopa/DDCI in patients with PD, conducted at outpatient consultations in 16 Spanish Movement Disorders centers.

Consecutive adult patients (≥18 years old) with PD, with an I-III Hoehn & Yahr stage during the ON periods (20), exhibiting levodopa-induced motor complications (motor fluctuations with and without dyskinesia), who were receiving opicapone at least during the preceding 3 months, were included in the study. This study encompassed a 12-month recruitment period. Patients attended a single visit in which they were informed and invited to participate in the study. Data on routine medical practice were retrospectively collected from patients’ medical records. This included clinical data before the treatment initiation with opicapone and those reported after 3 to 7 months of therapy. Given the non-interventional nature of the study, treatment decisions were not altered by the participation in the study. Study variables were extracted from patients’ medical records and recorded using a standardized case report form (CRF) to ensure consistency across centers. Only routinely documented variables were included, and ambiguous or inconsistently recorded data were excluded from the analyses.

The study was conducted in accordance with the ethical principles of the Declaration of Helsinki, Good Clinical Practice (GCP), and in compliance with European and national regulations. The Ethics Committee of Hospital Universitario de Navarra (Spain) approved the study, and written informed consent was obtained from all patients before their inclusion.

### 2.2 Study endpoints

The primary endpoint was the clinical change obtained in patients with PD after adding opicapone to the regular levodopa/DDCI regimen. The clinical change produced by opicapone was defined as the percentage of patients who reported motor fluctuations improvement without worsening dyskinesias (i.e., stable or improved dyskinesias) during the established period of treatment with opicapone. The change was evaluated using the Patient’s Global Impression of Change (PGI-C) scale (21), in which patients indicated whether they felt better (improved)/no change (stable)/worsening in both motor fluctuations and dyskinesias.

Secondary endpoints included the identification of the PD clinical phenotype that benefits most from opicapone treatment; the description of the effect of opicapone on certain PD symptoms; the clinical management of opicapone concerning concomitant treatments, the modification of levodopa doses after adding opicapone, the rate of opicapone therapy discontinuation; the evaluation of opicapone safety profile by collecting all adverse events (AEs) reported during opicapone treatment and treatment-related AEs; and the identification of demographic and clinical risk factors for developing dyskinesias after initiating treatment with opicapone.

### 2.3 Statistical analysis

We calculated that a sample size of 432 patients would be sufficient to estimate a percentage of 31.7% in the proportion of patients with PD experiencing improved clinical change during opicapone treatment as determined by PGI-C scores, with a 0.05 alpha and a precision of 4.5% in a bilateral contrast (assuming 5% data loss).

A descriptive statistical analysis was performed on the study variables. Measures of central tendency and dispersion (mean and standard deviation [SD], median and interquartile range [IQR]) were used to describe quantitative variables, while frequencies and valid percentages were used to describe qualitative variables; 95% confidence intervals (CI) were calculated. Data collected from medical records included patients’ age, sex, body mass index (BMI), and PD-related data, such as its severity and symptoms, age at onset, time of evolution of the disease, time from diagnosis to opicapone initiation, prior treatments, and history of dyskinesias. Group comparisons for categorical variables were made using Fisher’s exact, Wilcoxon-Mann–Whitney, or McNemar tests, as appropriate.

A logistic regression model was performed to study the association between the clinical change (PGI-C)/development of dyskinesias and each of the possible demographic or clinical factors (including prior treatments) of interest, starting with all variables that were significant in a first-step bivariate analysis (*p* < 0.002).

Missing data were not considered in the analyses, and no imputation was performed, reflecting the nature of routinely collected RWD. All hypothesis tests were bilateral; significance was considered at *p* < 0.05. All statistical analyses were conducted using the Statistical Package for the Social Sciences (SPSS) version 22.0 (SPSS Inc., Chicago, IL).

## 3 Results

### 3.1 Patient characteristics

Between August 2019 and May 2021, a total of 263 patients were enrolled in the study. Eighteen patients were excluded due to non-compliance with eligibility criteria; therefore, 245 patients were evaluable for statistical analyses. As shown in Table 1, the mean (SD) age was 67.7 (10.4) years, with a higher proportion of men (55.9%). The akinetic rigid phenotype was the most prevalent (41.9%), followed by the tremor-dominant (33.6%). Most patients with PD (63.9%) were in stage II of the Hoehn & Yahr scale. Concomitant clinical conditions unrelated to PD were reported in 62.4% of patients, the most frequent being hypertension (55.6%), dyslipidemia (39.9%), diabetes (21.6%), and neuropsychiatric history (13.1%; data not shown).

**Table 1:** General characteristics of study patients.

Characteristics	Value
Demographics	
Age (years), mean (SD) [N]	67.7 (10.4) [245]
Sex (male), n (%) [N]	137 (55.9) [245]
BMI (kg/m^2^), mean (SD) [N]	26.9 (4.7) [105]
Clinics of PD	
Time from diagnosis to treatment with opicapone (years), mean (SD) [N]	8.3 (5.3) [229]
Clinical phenotype, n (%) [N]	
Akinetic rigid	96 (41.9) [229]
Predominance of tremors	77 (33.6) [229]
Mixed or indeterminate	45 (19.7) [229]
PIGD	11 (4.8) [229]
Hoehn & Yahr stage	
I	10 (4.3) [230]
II	147 (63.9) [230]
III	73 (31.7) [230]
History of dyskinesias, n (%) [N]	
Yes	106 (43.8) [242]
No	136 (56.2) [242]

BMI, body mass index; PD, Parkinson’s disease; PIGD, postural instability and gait difficulties; SD, standard deviation; UPDRS, Unified Parkinson’s Disease Rating Scale.

On average (SD), patients started treatment with opicapone 8.3 (5.3) years after PD diagnosis. Almost all (98%) patients reported wearing-off motor fluctuations (as per established selection criteria), with a mean (SD) duration of daily OFF time of 143.3 (126.4) minutes. A previous history of dyskinesias was present in 43.8% of patients. Non-troublesome dyskinesias were reported by 29.0% of patients at the time of opicapone initiation, while troublesome dyskinesias were described in 3.3% (Supplementary Table 1).

Before the treatment with opicapone, the average (SD) daily levodopa dose was 620.7 (313.7) mg. In addition, 69.4% of patients had been treated with MAO-B inhibitors, 64.5% with dopamine agonists, and 11% with COMTi other than opicapone (Supplementary Table 2).

### 3.2 Treatment management

During opicapone treatment, levodopa dose was maintained stable in 63% of patients, increased in 18%, and reduced in 19%; on average, this reduction was of 210.7 (176.8) mg/day.

While receiving opicapone, 5.6 and 8.7% of patients stopped the treatment with dopamine agonists and MAO-B inhibitors, respectively. Most patients treated with antidepressants continued the treatment (96.5%; 55 patients).

### 3.3 Effectiveness of opicapone

**Figure 1:**
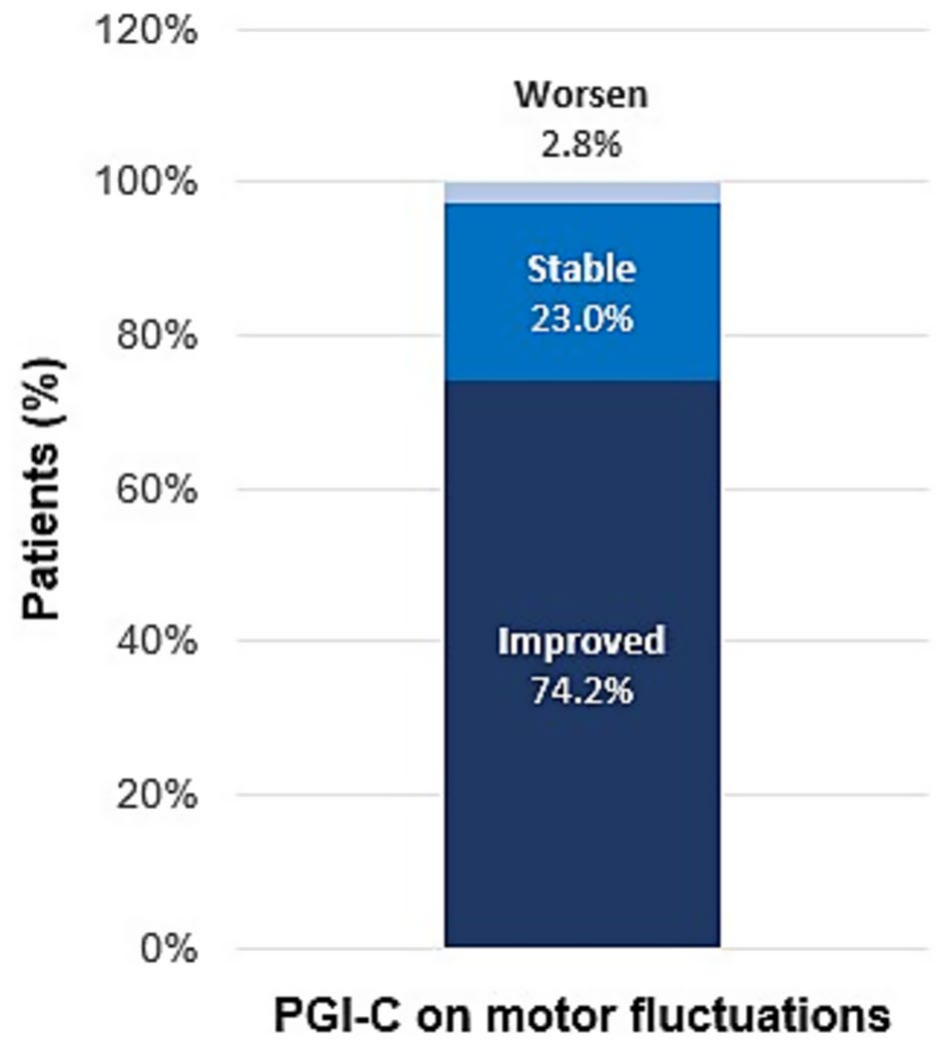
Clinical changes in motor fluctuations perceived by patients with PD treated with opicapone, according to the PGI-C scale. Percentage of patients who perceived the indicated clinical changes (improvement/stability/worsening) in motor fluctuations; PGI-C, Patient’s Global Impression of Change.

Data on the PGI-C was available for 178 patients (73% of 245 total patients). As shown in Figure 1, 74.2% (95% CI: 67.1–80.4%) of them reported improvements in motor fluctuations during the treatment with opicapone according to the PGI-C assessment. Indeed, 64.6% (95% CI: 57.1–71.6%) of patients noted clinically improved motor fluctuations without worsening dyskinesias (improved or stable).

No changes in motor fluctuations (stable) during opicapone treatment were reported by 23.0% of patients, while only 2.8% reported worsening of motor fluctuations (Figure 1). Overall (*n* = 178), 60.7% of patients reported stable dyskinesias, 23.6% improved, and 15.7% experienced worsening (not shown).

Among patients with a previous history of dyskinesias (*n* = 106), 38.7% (41 patients) did not show dyskinesia during treatment. Meanwhile, 84.6% (115 patients) of patients without a previous history of dyskinesias (*n* = 136) did not develop abnormal movements during treatment.

We also assessed the clinical change perceived in motor fluctuations and dyskinesias among patients with different PD phenotypes. For that analysis, we considered data from 166 patients. We found that improvement of motor fluctuations with stable/improved dyskinesias was not associated with a particular PD phenotype (*p* = 0.327; data not shown).

Subgroup analyses of patients’ demographic and clinical characteristics associated with PD clinical change are shown in Supplementary Table 3. Multivariate analyses revealed that the daily off time before opicapone treatment, the time with dyskinesias, and the complexity of motor fluctuations were independent predictive factors of clinical improvement in motor fluctuations with improved or stable dyskinesias. For instance, those patients with a lower daily off-time (in case of wearing-off motor fluctuations) before opicapone initiation, and those with a minimum time with dyskinesias or minimum complexity of motor fluctuations on the UPDRS, were more likely to perceive improvements in motor fluctuations without worsening dyskinesias.

**Figure 2:**
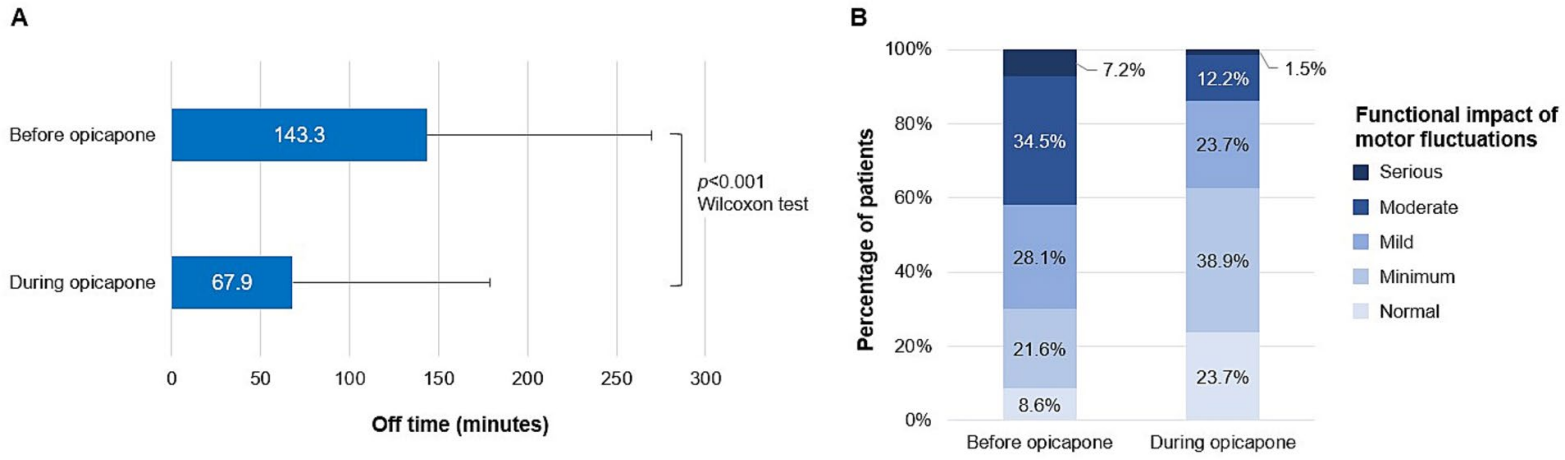
Impact of opicapone on motor fluctuations. **(A)** The daily off time (minutes), in case of wearing-off motor fluctuations, is shown as mean + standard deviation before starting treatment with opicapone and during treatment with opicapone. **(B)** Percentage of patients grading the functional impact of motor fluctuations according to the UPDRS part IV, before opicapone and during treatment with opicapone.

The effect of opicapone on PD symptoms was evaluated in the entire study cohort (N = 245). During the treatment with opicapone, the percentage of patients suffering from wearing-off motor fluctuations decreased to 61.6% (Table 2). In accordance, the mean (SD) daily off-time duration decreased up to 67.9 (111.2) minutes during opicapone treatment, compared to that before treatment initiation (143.3 [126.4] minutes; *p* < 0.001; Figure 2A). Based on item 4 of UPDRS Part IV, we found that the functional impact of motor fluctuations was reduced after opicapone treatment. Before treatment initiation, 69.8% of patients reported it as mild (28.1%), moderate (34.5%), or serious (7.2%). In contrast, during opicapone treatment, 37.4% of patients reported the functional impact of motor fluctuations as mild (23.7%), moderate (12.2%), or serious (1.5%; Figure 2B).

**Table 2:** Differences in the symptoms experienced by patients with PD after opicapone treatment initiation.

Symptoms of PD*	Patients, *n* (%)		*p*-value^a^
	Before opicapone	During opicapone	
Wearing-off motor fluctuations	240 (98.0)**	151 (61.6)	Not available***
Morning akinesia	88 (35.9)	59 (24.1)	<0.001
Non-troublesome dyskinesia	71 (29.0)	74 (30.2)	0.787
Non-motor fluctuations (pain, anxiety, distress)	53 (21.6)	37 (15.1)	0.010
Nocturnal akinesia	51 (20.8)	20 (8.2)	<0.001
Freezing of the gait	41 (16.7)	40 (16.3)	>0.999
Delayed-on motor fluctuations	25 (10.2)	13 (5.3)	0.010
Off dystonia	21 (8.6)	12 (4.9)	0.081
No-on motor fluctuations	16 (6.5)	7 (2.9)	0.027
Impulse control disorders	15 (6.1)	6 (2.4)	0.016
Falls	11 (4.5)	11 (4.5)	>0.999
Troublesome dyskinesia	8 (3.3)	15 (6.1)	0.146
Cognitive impairment	7 (2.9)	6 (2.4)	>0.999
Complex and unpredictable motor fluctuations	6 (2.4)	6 (2.4)	>0.999
Hallucinations and delusions	5 (2.0)	7 (2.9)	0.752

PD, Parkinson’s disease; SD, standard deviation. *PD symptoms were collected as presence/absence. Percentages may add more than 100% as each patient could present more than one symptom (multiple choice variable). **Although 5 patients did not select wearing-off motor fluctuation as a symptom in this questionnaire, all patients presented wearing-off motor fluctuations as they met the following inclusion criterion: “Patient with PD treated with levodopa + DDCI who presents wearing-off motor fluctuations with or without dyskinesias”. ***The *p*-value associated with this test could not be calculated because any patient (0%) had absence of wearing-off motor fluctuations before starting opicapone as per established selection criteria.

a McNemar test.

Besides, opicapone was able to alleviate most of the PD symptoms evaluated, including morning akinesia (35.9% vs. 24.1%; *p* < 0.001), non-motor fluctuations (21.6% vs. 15.1%; *p* = 0.010), nocturnal akinesia (20.8% vs. 8.2%; *p* < 0.001), delayed-on (10.2% vs. 5.3%; *p* = 0.010), and no-on motor fluctuations (6.5% vs. 2.9%; *p* = 0.027). The percentage of patients with impulse control disorder did not increase after initiating opicapone (6.1% vs. 2.4%; *p* = 0.016; Table 2).

As shown in Table 2, 89 (36.3%) patients experienced dyskinesias (troublesome and/or non-troublesome) during opicapone treatment. Of note, 79 of them (88.8%) already suffered from dyskinesias before starting treatment with opicapone. The mean (SD) percentage of daily time with dyskinesias did not show significant differences during opicapone treatment (25.1 [11.6] % vs. 28.5 [18.3] %; *p* = 0.454) as compared with the baseline evaluation.

We compared UPDRS data (*n* = 130) before opicapone initiation with those obtained after a mean (SD) time of 4.8 (3.6) months after opicapone initiation to determine changes in the disease stage. The mean score in UPDRS Part I remained stable in more than half (52.0%) of patients compared to before opicapone initiation. In contrast, scores on Parts II and III decreased in most (58.0 and 62.7%) patients, indicating improvements in activities of daily living and motor function. The mean (SD) score on the UPDRS I-III was 34.7 (13.6), showing a statistically significant decrease (*p* < 0.001) in 70.0% of patients during treatment with opicapone, compared to the assessment completed before starting the treatment. The mean score in Part IV also decreased significantly, confirming the improvement in motor complications (Table 3).

**Table 3:** Clinical change in the UPDRS after treatment with opicapone.

UPDRS	Total score mean (SD)		Change mean (SD)	*p*-value^a^
	Before opicapone	During opicapone		
Part I (*N* = 50)	2.9 (2.2)	2.5 (2.3)	−0.4 (1.5)	0.072
Part II (*N* = 50)	10.1 (6.4)	8.2 (5.4)	−2.0 (3.6)	<0.001
Part III (*N* = 110)	22.4 (9.5)	19.0 (10.7)	−3.4 (6.0)	<0.001
UPDRS I-III (*N* = 50)	34.7 (13.6)	28.3 (14.8)	−6.4 (11.1)	<0.001
Part IV (*N* = 107)	6.7 (3.8)	4.5 (3.3)	−2.2 (3.0)	<0.001

N, total of patients with available information of both UPDRS values (before and during opicapone) in medical records; SD, standard deviation; UPDRS, Unified Parkinson’s Disease Rating Scale.

a Wilcoxon test.

### 3.4 Tolerability

Overall, 12.2% (*n* = 30) of patients experienced any AE during the treatment with opicapone. A total of 30 AEs were considered related to opicapone, affecting 8.6% (*n* = 21) of patients. Among them, the most frequent AEs were dyskinesias (*n* = 9; 3.7%), hallucinations (*n* = 2; 0.8%), loose stools (*n* = 2; 0.8%), constipation (*n* = 2; 0.8%), insomnia (*n* = 2; 0.8%), and dizziness (*n* = 2; 0.8%; Table 4).

**Table 4:** Reported adverse events related to treatment with opicapone (*N* = 245).

Adverse events	Patients *n* (%)
Dyskinesia^a^	9 (3.7)
Constipation	2 (0.8)
Dizziness	2 (0.8)
Hallucinations	2 (0.8)
Insomnia	2 (0.8)
Loose stools	2 (0.8)
Anorexia	1 (0.4)
Asthenia	1 (0.4)
Cramps	1 (0.4)
Dopamine-dysregulation syndrome	1 (0.4)
Edema in the legs	1 (0.4)
Episodic confusional state	1 (0.4)
Gait unsteadiness	1 (0.4)
Nausea	1 (0.4)
Nightmares at opicapone initiation	1 (0.4)
Off-cervical dystonia	1 (0.4)
Psychiatric AEs: excitation	1 (0.4)

AEs, adverse events.

a Indicated as: dyskinesia (*n* = 2), increased dyskinesia (*n* = 1), disabling generalized choreic dyskinesias (*n* = 1), buccolingual dyskinesias (*n* = 1), choreic dyskinesias (*n* = 1), peak dyskinesias (*n* = 1), worsening of peak dyskinesias (*n* = 1), mild worsening of dyskinesias (*n* = 1).

Two (0.8%) patients reported severe AEs (SAEs). One of them reported hallucinations after 122 days from opicapone initiation, which was considered related to the treatment. The other patient developed acute respiratory failure, not related to opicapone.

## 4 Discussion

The present study provides evidence for the effectiveness and tolerability of opicapone as an add-on therapy in patients with mild to moderate PD in routine clinical practice in Spain and is in line with the results obtained in the randomized studies. A key finding of our study is that opicapone induces a similar motor benefit and safety profile across the different PD phenotypes.

In this real-world analysis, we observed clinical improvements in motor fluctuations in 74.2% of patients, and 64.6% reported improvements in motor fluctuations with stability/improvement on dyskinesias according to the PGI-C. These results are similar to those observed in the BIPARK I (13) and ADOPTION (16) clinical trials, RWD from the OPTIPARK study (15), and data reported in a review from 18 Spanish real-life studies with small patient cohorts (17). In these analyses, 72.1, 77.0, 76.9%, and 66–77% of patients, respectively, presented clinical improvement, according to their perceptions.

Together with clinical improvements in motor fluctuations, we demonstrated a 75.4-min daily off-time reduction during treatment with opicapone, which is consistent with the results observed in the BIPARK trials where opicapone was compared to placebo (13, 14). These results are also aligned with the available RWD of ≈200 patients from 18 patient series in Spain, in which the off time decreased between 72 and 84 min (17). The ADOPTION trial recently showed a reduction in absolute off time of 62.1 min in patients receiving opicapone 50 mg, which was significantly superior to the group receiving additional 100 mg of levodopa (−16.7 min) (16).

Furthermore, our study patients with PD reported a significant improvement in non-motor fluctuations, including pain, anxiety, and distress, during opicapone treatment. Similarly, the OPEN-PD, a study specifically designed to explore the effect of opicapone on non-motor symptoms, demonstrated an overall improvement in non-motor symptoms of the disease. In this study, significant enhancements in the mood/apathy, sleep/fatigue, gastrointestinal symptoms, and miscellaneous (that also assess pain) domains were observed (22). Likewise, the clinical practice OPTIPARK trial showed a reduction in non-motor symptoms, according to the Non-Motor Symptom Scale (NMSS), after 3 months of treatment with opicapone (15). More recently, the OASIS study provided further proof of the effect of opicapone on reducing PD-related sleep disorders and fatigue (23). A real-life observational analysis of data from 50 patients with PD from the King’s College Hospital Dubai also found improvement in non-motor burden as measured by the NMSS after 6 and 12 months of treatment with opicapone (24).

Besides the reduction of daily off time, an improvement in the severity of the “off” periods may affect the benefit registered in the PGI-C on motor fluctuations. Unfortunately, quantitative data (such as UPDRS III during off time) about off severity was not available as the UPDRS in ON and OFF conditions is not routinely recorded in clinical practice. However, item 4 of the UPDRS IV showed a qualitative improvement on this parameter, where 37.4% of patients qualified the functional impact of motor fluctuations as mild, moderate, or serious during opicapone treatment, as compared to 69.8% of patients before treatment initiation. Improvements in the UPDRS Part IV were also reported in the OPTI-ON study, in patients with PD experiencing OFF episodes who received opicapone and were followed up for 6 months (25). Although the off-period severity has been largely disregarded in PD treatment efficacy assessments, its impact on patients’ quality of life may be as relevant as the daily off time. The OPTIMO study’s data support the positive effect of opicapone on both parameters.

Of note, we found that the daily off time before opicapone treatment, the time with dyskinesias, and the complexity of motor fluctuations were predictive factors for the perception of clinical improvement in motor fluctuations without worsening dyskinesias. This observation is in line with the results of a post-hoc analysis of the BIPARK I/II studies in which patients with a shorter disease evolution (duration), lower PD severity, and shorter onset time of the motor fluctuations showed a trend for greater efficacy of opicapone (26). Moreover, an independent study identified longer disease duration and off time at baseline as predictive factors for opicapone treatment discontinuation (27). Accordingly, another post-hoc analysis of the BIPARK I/II studies and an Italian RWD study in 152 patients with PD showed that earlier patients, as opposed to later in their disease course, presented an even more favorable tolerability profile (28, 29). Even though opicapone is effective and safe at all stages and phenotypes of PD, these observations suggest that patients may benefit the most from introducing opicapone earlier in the course of the disease.

In addition to the clinical improvements reported for motor and non-motor fluctuations, upon opicapone treatment, patients showed a statistically significant reduction of 3.4 points in the UPDRS III (during on time), which reached the threshold of 2.3–2.7 points accepted as a clinically important difference (30). This suggests that the reduction in the daily off time induced by opicapone treatment may be accompanied by an improvement in the motor condition of the patient during on time. Overall, this ultimately contributes to patients’ experiences of daily living as assessed by the UPDRS Part II, where 58.0% of study patients showed an improvement upon opicapone treatment.

Adjuvant administration of opicapone increases levodopa bioavailability by 65% (31) and significantly prolongs its clinical effects (13, 14), which allows, in some cases, a reduction of the levodopa doses (18). The present study showed that the dose of levodopa was reduced in 19% of patients, and 82% did not require up-titration, suggesting a positive effect of opicapone on maintaining low levodopa doses.

The most common AE of opicapone is dyskinesia (32), which has often been associated with excessive dopaminergic stimulation during on periods due to enhanced levodopa exposure (33). In our study, opicapone did not significantly increase dyskinesias. Despite 36.3% of patients experiencing dyskinesia during opicapone treatment, most of them already suffered from it before starting the treatment, possibly due to the chronic nature of PD (with an evolution time of >8 years). Although data collected from medical records resulted in inconsistent reporting of dyskinesias as an AE, based on symptom reports, 15.4% (21 patients) of patients with no previous history of dyskinesias developed them during treatment. Aside from dyskinesias, other side effects reported were hallucinations, loose stools, constipation, insomnia, and dizziness. This tolerability profile is in harmony with the Spanish clinical practice series (17), a Portuguese RWD monocentric study (18), as well as with phase III BIPARK studies (13, 14, 34), in which treatment-related AEs mostly occurred within the first 2 months. Therefore, our results confirm that opicapone is generally well tolerated, with a low risk of AEs, even in a population with a long disease duration and prior medium-high doses of levodopa. Adding to our data, a meta-analysis by Xie L. and collaborators suggested that a brief course of opicapone led to an increased incidence of dyskinesia, whereas long-term treatment may not be associated with a higher risk in patients with PD (32), supporting its usefulness in managing PD motor fluctuations.

Limitations of this study include the absence of a control group and missing information on some variables due to the observational nature of the study and the fact that electronic medical records were originally designed for clinical care rather than research purposes. Another limitation is the reliance on patient-reported outcomes such as the PGI-C, which, although widely used in RWD studies, is inherently subjective and may be influenced by patient expectations or the physician-patient relationship. In addition, we could not include the estimated number of patients, and this may limit the traceability of the results obtained. Despite these limitations, to our knowledge, this is the most extensive study conducted to date in the real-world setting in Spain describing the effectiveness and tolerability of opicapone as adjuvant therapy to levodopa/DDCI for the treatment of PD. As this was a real-world study conducted within the Spanish healthcare system, the generalizability of these findings to other countries should consider differences in healthcare organization, clinical practice patterns, and patient management approaches.

In conclusion, this study expands the knowledge on the effectiveness and safety of opicapone in real-life patients with PD and complements clinical trial data. Our findings demonstrate that opicapone improves both motor and non-motor symptoms of the disease, thus enhancing patients’ perception of their PD condition. Additionally, the identified predictive factors for the improvement of motor fluctuations suggest that PD patients may benefit the most from the earlier introduction of opicapone. Opicapone also decreased the daily off time without significantly increasing dyskinesia, and reduced the functional impact during off time, making it an excellent adjunct treatment for PD motor fluctuations.

## Data Availability

The raw data supporting the conclusions of this article will be made available by the authors, without undue reservation.
