# Supplementary material for: Real-world data of opicapone in patients with Parkinson’s disease experiencing motor fluctuations: the OPTIMO study
Source: Front Neurol. 2026 Feb 19;17:1738500. doi: 10.3389/fneur.2026.1738500 (PMC12961986; doi:10.3389/fneur.2026.1738500)
Supplement: Supplementary file 1 [file Table_1.docx]

Supplementary Material

# Supplementary Tables

Supplementary Table 1. Symptoms of patients with PD at opicapone initiation

| **PD symptoms** | **Patients**  **n (%) N=245** | **Episodes**  **Mean (SD) [N]** |
| --- | --- | --- |
| Wearing-off motor fluctuations | 240 (98.0) | 2.9 (1.3) [139] |
| *Off-time duration (minutes), mean (SD) [N]* | *143.3 (126.4) [131]* | - |
| Morning akinesia | 88 (35.9) | 1.0 (0.2) [25] |
| Non-troublesome dyskinesias | 71 (29.0) | 2.3 (1.1) [10] |
| *Daily duration (%), mean (SD) [N]* | *29.2 (16.1) [25]* | - |
| Non-motor fluctuations | 53 (21.6) | 2.8 (1.3) [11] |
| Nocturnal akinesia | 51 (20.8) | 1.6 (0.7) [14] |
| Freezing of gait | 41 (16.7) | 3.6 (0.9) [5] |
| Delayed-on motor fluctuations | 25 (10.2) | 1.9 (1.2) [11] |
| Off dystonia | 21 (8.6) | 1.0 (0.0) [7] |
| No-on motor fluctuations | 16 (6.5) | 1.7 (0.6) [3] |
| Impulse control disorders | 15 (6.1) | Not available |
| Falls | 11 (4.5) | 3.0 (-) [1] |
| Troublesome dyskinesias | 8 (3.3) | Not available |
| *Daily duration (%), mean (SD) [N]* | *26.5 (16.9) [4]* | - |
| Cognitive impairment | 7 (2.9) | 0.0 (-) [1] |
| Complex and unpredictable motor fluctuations | 6 (2.4) | Not available |
| Hallucinations and delusions | 5 (2.0) | 3.0 (-) [1] |
| Other | 24 (9.8) | - |

PD, Parkinson’s disease; SD, standard deviation.

Supplementary Table 2. Prior treatments to opicapone

| **Treatments** | **Patients**  **n (%) [N]** |
| --- | --- |
| **Treatments for PD** |  |
| Levodopa | 244 (99.6) [245] |
| Levodopa/carbidopa | 188 (76.7) [245] |
| Levodopa/benserazide | 56 (22.9) [245] |
| Levodopa/carbidopa/entacapone | 13 (5.3) [245] |
| L-dopa | 2 (0.8) [245] |
| Duodopa | 1 (0.4) [245] |
| *Daily dose of levodopa (mg), mean (SD) [N]* | *620.7 (313.7) [227]* |
| *Duration of levodopa treatment (months), mean (SD) [N]* | *58.8 (51.6) [171]* |
| MAO-B inhibitors | 170 (69.4) [245] |
| Safinamide | 107 (62.9) [170] |
| Dopamine agonist | 158 (64.5) [245] |
| COMTi (other than opicapone) | 27 (11.0) [245] |
| Entacapone | 14 (51.9) [27] |
| Levodopa/carbidopa/entacapone* | 13 (48.1) [27] |
| Other | 13 (5.3) [245] |
| **Other treatments** |  |
| Antidepressants | 64 (26.1) [245] |
| Antipsychotics | 9 (3.7) [245] |

COMTi, Catechol-O-methyl transferase inhibitors; MAO-B, type-B monoamine oxidase; PD, Parkinson’s disease; SD, standard deviation.

*It is also included in levodopa-related treatments.

Supplementary Table 3. Patient characteristics associated with clinical improvements in motor fluctuations and improved/stable dyskinesias

| **Bivariate analysis^¥^** | | **Perception of clinical change**  **(improved MF + improved/stable D)** | | | ***p*-value** |
| --- | --- | --- | --- | --- | --- |
|  |  | **Yes** | **No** | |  |
| **Off-time** (minutes), mean (SD) | | 142.1 (107.6) | 213.9 (166.0) | | 0.042^a^ |
| **History of dyskinesias,** n (%) | | | | | |
| No | | 70 (69.3) | 31 (30.7) | | 0.111^b^ |
| Yes | | 42 (56.8) | 32 (43.2) | |  |
| **Treatment before opicapone,** n (%) | | | | | |
| DA | No | 42 (65.6) | 22 (34.4) | | 0.871^b^ |
|  | Yes | 73 (64.0) | 41 (36.0) | |  |
| MAO-B inhibitors | No | 39 (72.2) | 15 (27.8) | | 0.176^b^ |
|  | Yes | 76 (61.3) | 48 (38.7) | |  |
| COMTi (other than opicapone) | No | 103 (65.6) | 54 (34.4) | | 0.472^b^ |
|  | Yes | 12 (57.1) | 9 (42.9) | |  |
| **UPDRS** | | | | | |
| Time with dyskinesias, n (%) | Normal | 52 (72.2) | 20 (27.8) | | 0.012^b^ |
|  | Minimum | 6 (35.3) | 11 (64.7) | |  |
|  | Mild | 15 (83.3) | 3 (16.7) | |  |
|  | Moderate | 3 (60.0) | 2 (40.0) | |  |
| Complexity of motor fluctuations, n (%) | Normal | 22 (62.9) | 13 (37.1) | | 0.050^b^ |
|  | Minimum | 23 (57.5) | 17 (42.5) | |  |
|  | Mild | 25 (86.2) | 4 (13.8) | |  |
|  | Moderate | 8 (80.0) | 2 (20.0) | |  |
| **Multivariate analysis** |  | **OR** | **95% CI** | | ***p*-value^c^** |
| **Off-time** |  | 0.99 | 1.0 – 1.0 | | 0.031 |
| **Time with dyskinesias*** | Minimum | 0.03 | 0.0 – 0.2 | | 0.001 |
|  | Mild | 0.70 | 0.1 – 6.0 | | 0.722 |
|  | Moderate | 0.29 | 0.0 – 7.6 | | 0.376 |
| **Complexity of motor fluctuations*** | Minimum | 0.09 | 0.0 – 0.7 | | 0.057 |
|  | Mild | 0.58 | 0.0 – 8.3 | | 0.700 |
|  | Moderate | 0.62 | | 0.0 – 22.4 | 0.776 |

CI, confidence interval; COMTi, Catechol-O-methyl transferase inhibitors; D, dyskinesias; DA, dopamine agonist; MAO-B, type-B monoamine oxidase; MF, motor fluctuations; OR, odds ratio; SD, standard deviation; UPDRS, Unified Parkinson’s Disease Rating Scale.

^a^Wilcoxon Mann-Whitney test, ^b^Fisher’s exact test, ^c^Multiple logistic regression analysis

^¥^Only the data of those variables that were significant in the bivariate analysis (*p*<0.200) or the multivariate analysis (*p*<0.05) are shown.

*Reference category: normal
